# Translation, content validity, and preliminary psychometric validation of the *Existential Distress Scale* in a Spanish non-clinical population

**DOI:** 10.3389/fpsyg.2026.1771082

**Published:** 2026-03-25

**Authors:** Susana Martínez Rodríguez, Clara Molinero Caparrós, Gema Pérez-Rojo

**Affiliations:** 1 Universidad Francisco de Vitoria, Pozuelo de Alarcón, Spain; 2 Universidad CEU San Pablo, Madrid, Spain

**Keywords:** existential distress, loneliness, meaning in life, self-worth, validation

## Abstract

**Purpose:**

This paper presents the adaptation, content validity assessment, and initial psychometric evaluation of the Existential Distress Scale (EDS) in a Spanish non-clinical sample. Exploratory factor analysis (EFA) and confirmatory factor analysis (CFA) were performed in independent samples to evaluate the scale’s internal structure. This scale evaluates the presence of existential distress based on three constructs: loneliness, self-worth and meaning in life.

**Methods:**

For the adaptation to Spanish, a translation and retro-translation was carried out with the judgment of seven experts. For the psychometric evaluation of the instrument, a cross-sectional study was conducted using two independent samples, comprising 197 participants for the exploratory factor analysis and 150 for the confirmatory factor analysis.

**Results:**

Content validity was examined, and criterion-related validity was preliminary verified, with effect sizes indicating partial convergent validity. Exploratory and confirmatory factor analyses supported a three-factor structure and provided initial evidence of adequate factorial validity.

**Conclusion:**

The results provide initial evidence of promising psychometric properties in a Spanish non-clinical sample. The resulting tool provides a brief and operational measure of a complex construct relevant to understanding human suffering.

## 1 Introduction

The existential position of an individual emerges in the first relationship with another person, from the earliest moments of life (Martorell, 1986). Individuals with mental health problems are more likely to have a negative existential position, often accompanied by feelings of anguish or distress (Budiša, et al., 2012).

The existential position is therefore the consequence of considerations or judgements on the meaning and value of life (Lo, 2018), and the term existential distress may be applied when doubts rise about the meaning and value of one’s own life. This consideration generally changes over the course of the life cycle of an individual, especially at moments of crisis in one’s life (Erikson, 1985). The unsatisfactory resolution of a life crisis may result in this existential distress or anguish, posing a risk to the psychic wellbeing of the individual and ultimately lead to a crisis of meaning in life. Thus, perceived meaninglessness of life can constitute the clinical basis for several forms of psychological dysfunction (García-Haro et al., 2018).

It was not until after the mid-19th century that existential distress began to be considered a medical or psychopathological issue. In 1884, Kierkegaard distinguished existential distress from other emotional constructs such as fear, fright, panic, terror or shivering (Pizarro, 2011). Since then, existential distress has been defined as “a basic disposition of existence that becomes evident in boundary situations” (Jaspers, 1993, p. 131). That is, in difficult situations, the person becomes aware of the existential position that are in. Lo et al. (2016) conceptualize existential distress as a multidimensional experience of intense psychological suffering related to the loss of identity, connection with others and meaning of life. It is a dimensional construct that can manifest itself to varying degrees in the general population (Chavez-Baldini et al., 2024).

Existential concerns, such as isolation and the absence of meaning in life, are associated with certain psychological diagnoses, for example borderline personality disorder (Menzies et al., 2025). An association has been found between the experience of existential distress and clinical symptomologies of depression (Baert et al., 2020) and anxiety (Hooker and Bekelman, 2015). The management of existential distress and suffering is therefore crucial to mental health.

Existential distress also appears to be associated with existential loneliness, characterized by feelings of emptiness, disconnection and meaninglessness in life. This loneliness can be a consequence of feelings of disconnection with the world (McKenna-Plumley et al., 2023), accompanied by negative emotions and appraisals that emerge from adolescence (Garnow et al., 2024). Factors associated with existential loneliness include the avoidance of attachment (Helm et al., 2020), low frustration tolerance (Salical and Guler, 2022) and social withdrawal (Galanaki et al., 2023). Early therapeutic intervention can be effective in preventing the appearance of suicidal ideation and depression (Helm et al., 2020).

Feelings of low self-esteem or self-worth is also related to the presence of existential distress. Individuals with low self-esteem are more likely to experience existential distress given that self-esteem is considered a protective factor (Bergman and Bodner, 2022). Ryff (1989) notes that self-esteem is a key factor in the wellbeing of an individual. Leading a life of meaning and purpose is closely associated with higher levels of self-esteem (Stempelová and Čmáriková, 2004).

Existential distress thus comprises three psychological constructs that are distinct but interrelated. An interpersonal construct related to the experience of loneliness (Wein, 2012), an intrapersonal construct described as diminished self-validation (Tomasdottir et al., 2016), and a cognitive-existential construct such as the perception of a lack of meaning or purpose (Georgiou and Kleftaras, 2024).

Psychological interventions in cases of existential distress have been undertaken in clinical populations at the end of life, for example those with diagnosed with cancer or receiving palliative care (Lo, 2018; Niles et al., 2021). Psychotherapy among these patients generally focusses on existential fears, identity and uncertainty about the future, in order to alleviate the feelings of demoralization they may feel (Vehling and Philipp, 2018). In this population, it is essential to focus on the meaning and purpose of life by working on social relationships, self-esteem, religious faith and the degree and impact of physical suffering (Lo, 2018).

To assess the presence of existential distress in adults, an appropriate instrument is required to identify its essential components. The *Existential Distress Scale* (EDS) was developed after an exhaustive review of various published and validated English-language scales related to meaning in life. The review also identified recurrent items related to loneliness and self-esteem. The tools examined included the *Existential meaning scale* (Lyon and Younger, 2005), the *Rosenberg Self-esteem Scale* (Rosenberg, 1965), and the *UCLA Loneliness Scale* (Russell, 1996), among others.

In the development of the original EDS a total of 22 items were proposed, which were refined and reduced over the course of meetings with experts in palliative care, resulting in a final version of 10 items (Lo et al., 2016). The authors propose that feelings of loneliness (items 1–3), low self-worth (items 4–7) and meaninglessness (items 8–10) are the essential components of existential distress among adults. The final score indicates the presence and severity of this distress, and also concisely evaluates using simple items the three subdimensions: sense of connection with others, self-worth and meaning in life.

The determination of the validity of the instrument and its factor loadings was based on its application with a sample of 21 oncology patients (Lo et al., 2016). An exploratory factor analysis grouped the 10 items into a single factor. The authors suggested expanding the sample to a minimum of 60 individuals in order to provide the necessary statistical power to validate the instrument in accordance with the guidelines proposed by Bujang et al. (2018).

Given that previous validity testing was limited and conducted in a non-Spanish clinical population, the main objective of this study was to adapt EDS into Spanish and to conduct a preliminary psychometric validation in a Spanish non-clinical sample. The secondary objectives were to analyse the factorial structure of the instrument, assess its content validity, and examine its internal consistency.

## 2 Materials and methods

### 2.1 Participants

Two independent samples were taken in this study. The first sample was taken between February and May 2024, and the second during December 2025.

Both Samples were selected following the same inclusion and exclusion criteria. The inclusion criteria required participants to be over 18 years of age and Spanish nationality. The first sample was drawn from the general population, whereas the second sample consisted of university students. As an exclusion criterion, participants currently undergoing psychopharmacological treatment were not included in the study.

Initially the *first sample* consisted of 302 individuals. Of these, 75 were excluded due to incomplete data, and a further 30 were excluded for being under prescribed psychopharmacological treatment by a mental health professional. Finally, this first sample included 197 individuals, with a mean age of 39.19 (*SD*_*age*_ = 13.5). Of these, 26 % were men (*M_*age*_* = 44.47; *SD*_*age*_ = 12.75) and 74% were women (*M_*age*_* = 37.35; *SD_*age*_* = 13.34).

Regarding the sociodemographic characteristics of the first sample, about marital status, 38.6% are single, 44.7% are married, 7.6% are in a civil partnership; 6.6% are separated or divorced, 0.5 and are widowed and a 2% prefer not to answer. The 91.4% of the people included in the study have university or postgraduate education, and 8.1% have completed secondary education. Only 0.5% have primary education. Regarding employment status, 55.3% are employed by others and 14.3% are self-employed. 22.8% are students, and a small proportion, 4%, do not work because they are retired, unable to work or unemployed.

The analyses showed that there were no significant differences in the sample in terms of sex (*U* = 3070, *p* = 0.062) and educational level (*U* = 1489, *p* = 0.857).

The *second sample* consisted at first of 187 people. Thirty-seven were excluded because they were not Spanish and/or were prescribed psychopharmacological treatment. 150 individuals made up the second sample, with a mean age of 19,6 (*SD_*age*_* = 3.48). Of these, 42 % were men (*M_*age*_* = 20.60; *SD_*age*_* = 4.80) and 58% were women (*M_*age*_* = 19.30; *SD_*age*_* = 2).

All of them were university undergraduates, with 80% being single, 11.3% married or in a civil partnership. One person was divorced and the rest (8%) preferred not to answer.

In this second sample, when differentiating by sex, a significant difference is observed (*U* = 2110, *p* = 0.018), although the effect size is small (*z* = −2.36).

The first sample size adequacy for the exploratory factor analysis was determined according to the criteria proposed by Bujang et al. (2018), while the sample size for the confirmatory factor analysis (the second sample) was considered sufficient based on recommendations for CFA models of low to moderate complexity (Hair, 2009).

### 2.2 Materials

An assessment protocol was created to collect the data related to the variables of the study and included:

*Sociodemographic questionnaire:* An *ad hoc* instrument to collect data on age, sex, country of birth, education level, occupation, marital status and current treatment with psychopharmacological medication.

*Existential Distress Scale Spanish version* (EDS-S; *Escala de Angustia Existencial):* As with the original English language version, the EDS-S consists of three subscales and 10 items in total measuring three dimensions: Loneliness, Low Self-worth, and the Experience of Meaninglessness. The items are rated using a 5-point Likert-type scale from 0 = no distress to 4 = unbearable distress. All scores are direct, meaning that higher scores indicate greater levels of distress. The “Loneliness” subscale, with three items, refers to how a person perceives their connection with others; the “Low Self-worth” subscale, with four items, refers to feelings of self-worth and perceived valuation by others; the “Experience of Meaninglessness,” with three items, refers to the evaluation of meaning or purpose in life.

The total score of the scale ranges from 0 to 40 points, with higher scores indicating a greater degree of perceived existential distress.

For this study, the instrument was translated from the English version (Lo et al., 2016) with a Cronbach’s alpha of 0.86. The Spanish language version of the scale had a Cronbach’s alpha of 0.93 for the first sample and 0.92 for the second sample.

*UCLA Loneliness scale*, version 3, Spanish version (Russell, 1996, adapted by Vázquez and Jiménez, 1994).

This is a 10-item instrument that measures the feelings of loneliness of an individual. The items are scored using a Likert-type scale from 1 to 4 with a minimum score of 10 and a maximum of 40. The cut-off points established by the authors rated scores under 20 points as indicating severe loneliness, 20–30 points indicating moderate loneliness. The Cronbach’s alpha of the original scale was 0.94, 0.89 for the first sample and 0.85 for the second.

Purpose in Life Test short version (PIL-10), derived from the original instrument developed by Crumbaugh and Maholick (1969) and later adapted into a 10-item Spanish version by García-Alandete (2014).

This is a 10-item test with responses in a Likert-type scale of 1–7 developed to assess the feelings of meaningfulness of life of an individual based on two factors: Life Satisfaction and Meaning in Life, and Goals and Purposes. The test has a Cronbach’s alpha of 0.86. The scoring is direct, ranging from between 10 and 70 points. There are no cut-off points and the higher the score the greater the feelings of Meaning in Life of the person. The Cronbach’s alpha in the first sample was 0.92 and 0.86 for the second.

*Rosenberg Self-Esteem Scale (*RSES*)*, Spanish version (Rosenberg, 1965; translated and validated by Martín-Albo et al., 2007).

The scale presents a 10-item self-report scale developed to assess global self-esteem, understood as the individual’s overall evaluation of self-worth. Responses are provided on a 4-point Likert type scale, from 1 (strongly disagree) to 4 (strongly agree). There are five items negatively worded (2, 5, 6, 8, and 9) and are reverse score. The scoring is obtained by summing all items after reverse coding, ranging from 10 to 40 points. Hire scores indicate higher levels of local self-esteem. The Cronbach’s alpha in the second sample was 0.83.

### 2.3 Procedure

The study was approved by the university ethics committee and conducted in accordance with the ethical standards outlined in the Declaration of Helsinki.

#### 2.3.1 Phase 1: translation and cultural adaptation of the instrument

The translation process was carried out in two sub-phases. First, the English version of the EDS was translated by the university’s institutional translation service. Subsequently, a back-translation was performed by a bilingual professor, PhD in Psychology, whose native language is Spanish. The research team then synthesized and harmonized both versions to produce a final translated version, which was subsequently submitted to a panel of experts for evaluation.

#### 2.3.2 Phase 2: panel of experts

In this phase, seven experts were recruited based on their academic background, professional experience and qualifications. The panel consisted of three Doctors of Psychology specializing in research methodologies, a psychologist specializing in methodology, two doctors of psychotherapy specializing in grief, forensic psychology and trauma intervention, and a psychotherapist expert in grief and emergency care. Existential issues such as the meaning of life, suffering, and coping with loss are present in these fields of intervention (Ivers et al., 2024; Neimeyer, 2019). While the panel provided extensive expertise in the psychological mechanisms of existential distress, the specific clinical phenomenology of end-of-life care in oncology settings was represented indirectly through the expertise in grief and trauma. These experts were invited to participate and provided with a document which outlined the research objectives, the purpose of the EDS and the role of the panel of experts. The panel was asked to assess each item considering three dimensions: clarity, relevance and coherence (Pedrosa et al., 2013).

**FIGURE 1:**
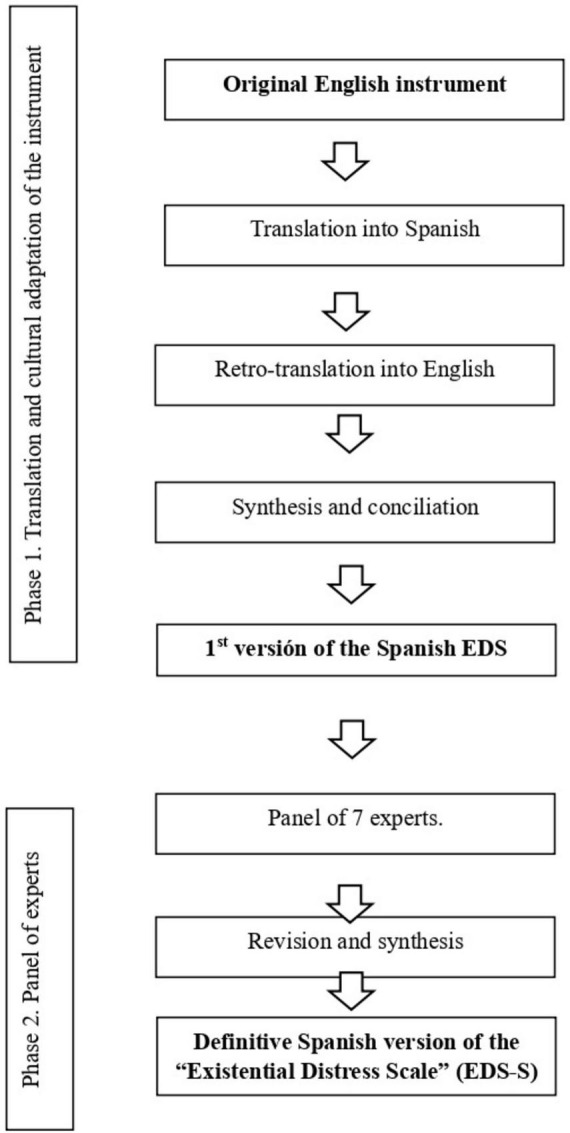
Flow diagram of the translation and adaptation process for the “Existential Distress Scale” into Spanish.

Each item was assessed based on these three dimensions (clarity, relevance and coherence) using a 4-point Likert-type scale: 1 = not clear, 2 = somewhat clear, 3 = fairly clear, 4 = very clear, 1 = not relevant, 2 = somewhat relevant, 3 = fairly relevant, 4 = very relevant, 1 = not coherent, 2 = somewhat coherent, 3 = fairly coherent, 4 = very coherent (Pedrosa et al., 2013). Each expert rated each item accordingly and added comments if considered necessary. Subsequently, the authors of the study then analyzed and discussed any discrepancies or ambiguities and produced the first version of the instrument in Spanish. This process is illustrated in Figure 1.

The content validity of the questionnaire was estimated through content validity coefficients for each item (CVC), calculated as the ratio between the sum of the scores given by each expert and the total number of participating experts, and for the entire scale (calculated as the total sum of all CVCs divided by the total number of items in the instrument), also accounting for inter-rater error (CVCt) (Hernández-Nieto, 2002).

#### 2.3.3 Phase 3: application of the questionnaire for the study of validity and reliability

Two independent surveys were conducted at two different times. In both cases, data was collected using the Qualtrics platform. A non-probabilistic snowball method was used in both cases, targeting the general population in the first case and university students in the second. Before starting the questionnaire, all participants were informed of the purpose of the research through an informed consent form, which assured them of anonymity and confidentiality in accordance with the Spanish Data Protection Act.

The questionnaires were administered in the same order for all participants: an ad hoc sociodemographic questionnaire, the *Existential Distress Scale, Spanish version*, the *UCLA Loneliness Scale*, and the *Purpose in life test*, for the first sample, adding the *Rosenberg Self-Esteem Scale* for the second sample.

### 2.4 Data analysis

Once the translation and linguistic and cultural adaptation of the instrument had been completed, the expert panel review was evaluated according to the Content Validity Coefficient (CVC) and the total CVC (Hernández-Nieto, 2002).

To verify whether the sample followed a normal distribution, the Kolmogorov-Smirnov test was applied. The results (*p* < 0.05) indicated that the sample did not follow a normal distribution, and therefore non-parametric tests were used.

A descriptive analysis of the sample was performed (mean, standard deviation, skewness, and kurtosis indices). Internal consistency of the items was assessed using Cronbach’s alpha and Mc Donald’s omega.

To examine the factorial structure of the EDS-S, an exploratory factor analysis (EFA) was conducted using the first sample. The unweighted least squares (ULS) extraction method was applied due to the nor-normal distribution of the variables, as data did not meet the assumption of multivariate normality. An Oblimin rotation with Kaiser normalization was selected given the expected interdependency among factors.

Subsequently, a confirmatory factor analysis (CFA) was performed using an independent second sample to test and confirm the factorial structure derived from de EFA, applying the same Oblimin rotation with Kaiser normalization.

To examine convergent and divergent validity, Spearman’s correlations were calculated between the subscale scores of the *Existential distress scale, Spanish version* and the subscale scores of the *UCLA Loneliness Scale*, the *Purpose in Life test* and the *Rosenberg Self-Esteem Scale*. All correlational analyses were conducted using data obtained for the second sample.

Data analysis was performed using SPSS version 29, and R version 4.5.1.

## 3 Results

### 3.1 Content validity analysis of the test

As shown in Table 1, the content validity indicator exceeded a score of 0.90 for all items, indicating excellent validity and agreement, while also accounting for inter-rater error (Hernández-Nieto, 2002). All items were found to be clear, relevant, and consistent with the construct intended to be measured.

**TABLE 1:** Content validity indicators.

Item	Global CVC	CVCt (without probability of error between experts)	Validity and concordance
1	0.98	0.98	Excellent ( > 0.90)
2	0.98	0.98	Excellent ( > 0.90)
3	0.96	0.96	Excellent ( > 0.90)
4	0.93	0.93	Excellent ( > 0.90)
5	0.96	0.96	Excellent ( > 0.90)
6	0.92	0.92	Excellent ( > 0.90)
7	0.95	0.95	Excellent ( > 0.90)
8	0.98	0.98	Excellent ( > 0.90)
9	1	1	Excellent ( > 0.90)
10	1	1	Excellent ( > 0.90)
Mean translated EDS-S	0.97	0.97	Excellent ( > 0.90)

With these results and considering the opinions of the panel of experts, minor modifications were in the semantics and syntax of the items. As shown in Table 1, these modifications were:

1. Syntactic modifications: adding the more formal form of “you” (*“usted”*) to items 5, 6, 7, and 10
2. Semantic modifications:

The term “afflicted” was substituted for “distressed” throughout the questionnaire, considered more understandable for participants.

In item 1, this change was reflected in the substitution of “afflicted” for “distressed”

In item 2, the expression “when you have gone” (“*cuando se haya ido*”) was substituted for “when you die” (*“cuando fallezca”)*.

The final version of the scale included 10 items divided into three subscales analogous to the original version: three items related to Loneliness (items 1, 2, and 3), four items related to Low Self-worth (items 4, 5, 6, and 7), and three items related to Experience of Meaninglessness (items 8, 9, and 10).

Table 2 presents the syntactic and semantic modifications made to the items during the process of translation, retro-translation, review by the panel of experts, and subsequent syntactic and semantic adaptation.

**TABLE 2:** Syntactic and semantic modifications and cultural adaptions of the EDS items.

Translation and retro-translation process			
Syntactic modifications			
Original	Translation	Retro-translation	Version 1 EDS-S
**Scale**. That you are all alone?	Are you alone?	That you are alone?	Are you completely alone?
**Semantic modifications**			
**Original**	**Translation**	**Retro-translation**	**Version 1 EDS-S**
**Instructions**. How distressed have you been by the thought:	How afflicted are you by the thought:	How distressed have you been by the thought:	Do you feel distress at the thought…?
**Panel of experts and revision**			
**Semantic modifications**			
**Original**	**Judgment of experts**	**Final version of the EDS-S**	
2. Will no one miss you when you have gone?	Substitute the expression “when you have gone” for “when you die”	Will no one miss you when you die?	
4. Are you or will you be a burden to others?	Use the more formal term “Usted”	Are you or will you be a burden to others?	
5. Do you have nothing to offer others?	Use the more formal term “Usted”	Do you have nothing to offer others?	
6. Do you not matter?	Use the more formal term “Usted”	Do you not matter?	
7. Are you worthless?	Use the more formal term “Usted”	Are you worthless?	
10. Do you have nothing to live for?	Use the more formal term “Usted”	Do you have nothing to live for?	

### 3.2 Descriptive analysis of the EDS-S

As shown in Table 3, the items of the EDS-S exhibit positive skewness values below 3 points and kurtosis values around zero. The item-total correlation is above 0.60. Standard deviations range between 0.94 and 1.18, indicating an adequate level of variability. Regarding the corrected item-total correlation, values are above 0.60.

**TABLE 3:** Descriptive statistics of the EDS-S items.

ITEM	M	DT	Asymmetry	Kurtosis	Correction of items-Total corrections	Frequencies of response				
						1 (No distress)	2 (Mild distress)	3 (Moderate distress)	4 (Great distress)	5 (Unbearable distress)
EDS-S 1	1.93	1.09	1.067	0.226	0.65	90	60	22	20	5
EDS-S 2	1.61	0.94	1.453	1.151	0.62	125	38	21	12	1
EDS-S 3	1.96	1.06	0.75	−0.644	0.78	90	49	35	22	1
EDS-S 4	2.05	1.07	0.714	−0.574	0.68	78	60	32	25	2
EDS-S 5	1.85	1.11	1.176	−0.263	0.77	104	50	16	23	4
EDS-S 6	1.89	1.12	1.072	−0.091	0.83	102	44	27	19	5
EDS-S 7	1.88	1.18	1.076	−0.122	0.87	110	35	23	24	5
EDS-S 8	1.92	1.11	1.026	−0.041	0.84	96	52	22	23	4
EDS-S 9	1.77	1.13	1.387	0.808	0.78	117	40	15	19	6
EDS-S 10	1.56	1.05	1.775	1.835	0.76	144	21	10	19	3

### 3.3 Exploratory factor analysis

In the original study by Lo et al. (2016), the presence of a single factor was suggested, accounting for 51% of the variance. The authors suggested that increasing the sample size to a minimum of 60 would provide sufficient power to conduct a validation study.

In the present study, an Exploratory Factor Analysis (EFA) was conducted on data from a sample of 197 participants to examine the latent structure of the Spanish-translated version of the instrument. Sampling adequacy was assessed (KMO = 0.98), and Bartlett’s test of sphericity was significant (χ^2^ = 1497.64; *p* < 0.001), supporting the suitability of data for factor analysis. Factor extraction was performed using unweighted least squares (ULS) based on a Pearson correlation matrix. An Oblimin rotation method with Kaiser normalization was used, suppressing loadings equal to or below 0.30, given the correlations among the factors.

The results indicate the presence of three factors explaining 78.5% of the variance. As shown in Table 4, the pattern matrix reveals three factors corresponding to the three interpretable factors corresponding to the theoretically proposed dimensions: Loneliness (items 1–3), Low Self-worth (items 4–7), and Experience of Meaninglessness (items 8–19).

**TABLE 4:** EFA pattern matrix.

Item	Factor		
	1	2	3
EDS-S 1 loneliness	0.105	−0.107	**0.500**
EDS-S 2 loneliness	0.032	0.013	**0.685**
EDS-S 3 loneliness	−0.047	0.024	**0.997**
EDS-S 4 low self-worth	**0.499**	−0.197	0.029
EDS-S 5 low self-worth	**0.926**	0.079	0.005
EDS-S 6 low self-worth	**0.607**	−0.071	0.257
EDS-S 7 low self-worth	**0.544**	−0.300	0.123
EDS-S 8 meaninglessness	0.269	−**0.665**	0.026
EDS-S 9 meaninglessness	0.011	−**0.971**	−0.055
EDS-S 10 Meaninglessness	−0.059	−**0.815**	0.125

Bold values indicate the primary factor loadings for each item in the exploratory factor analysis.

The final Spanish version consists of 10-item scale with high internal consistency, α = 0.93, ω = 0.93, with three first-order factors. Factor 1, named Low Self-worth, has an α of 0.88, ω = 0.89, while Factor 2, named Meaninglessness, has an α of 0.92, ω = 0.91. Lastly, Factor 3, named Loneliness, has an α of 0.80 and an ω = 0.81. Inter-factor correlations are shown in Table 5.

**TABLE 5:** Factor correlation matrix*.

Correlations	Factor 2	Factor 3
Factor 1	−0.71	0.76
Factor 2		−0.63

*Extraction method: unweighted least squares. *Rotation method: Oblimin with Kaiser normalization.

### 3.4 Confirmatory factor analysis

A Confirmatory Factor Analysis (CFA) was conducted to test the three-factor model identified in the EFA. The model showed an acceptable fit to the data, χ^2^ = 43.27 *p* < 0.001. Incremental fit indices indicated very good model fit, with a CFI = 0.997 and a TLI = 0.996. RMSEA was 0.049, with a 90% confidence interval ranging from 0.000 a 0.083, indicating good error of approximation. The SRMR value was 0.04, supporting the adequacy of the model fit.

All variables loaded significantly in their respective latent factors (*p* < 0.001), with standardized factor loadings ranging from moderate to high. The latent factors were allowed to correlate, and moderate to strong correlations were observed among them, reflecting theoretical relatedness of the constructs assessed. Standardized and unstandardized factor loadings for the three-factor model are reported in Table 6.

**TABLE 6:** Factor loadings for the three-factor CFA model.

Item	Factor	std all	se	*p*-value
EDS-S 1	Loneliness	0.521	0.070	<0.000
EDS-S 2	Loneliness	0.777	0.049	<0.000
EDS-S 3	Loneliness	0.931	0.037	<0.000
EDS-S 4	Low Self-worth	0.774	0.042	<0.000
EDS-S 5	Low Self-worth	0.769	0.038	<0.000
EDS-S 6	Low Self-worth	0.928	0.022	<0.000
EDS-S 7	Low self-worth	0.943	0.020	<0.000
EDS-S 8	Experience of meaninglessness	0.887	0.024	<0.000
EDS-S 9	Experience of meaninglessness	0.952	0.018	<0.000
EDS-S 10	Experience of meaninglessness	0.906	0.025	<0.000

Std all, standardized factor loading; se, Standard error. All loadings were statistically significant (*p* < 0.000). The CFA was estimated using the WLSMV estimator in R.

**FIGURE 2:**
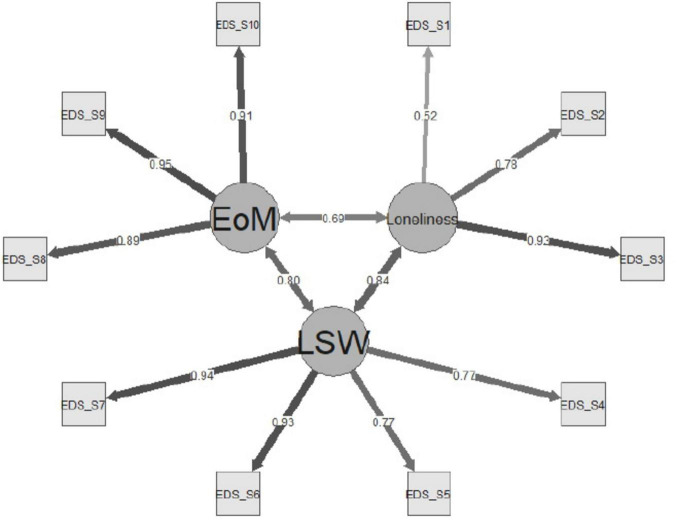
CFA path diagram. EOM, Experience of Meaningless Factor; Loneliness, Loneliness factor; LSW, Low-Self Worth Factor; EDS-S number, DS-S Item.

Figure 2 presents the path diagram of the three factor CFA model.

After establishing the factorial structure, measurement invariance across sex was tested via multi-group confirmatory factor analysis. First, the configurational model showed an adequate fit (CFI = 0.955; RMSEA = 0.096; SRMR = 0.048), indicating that the factor structure is equivalent in men and women. Subsequently, the metric model showed minimal variations from the configural model (ΔCFI = −0.002; ΔRMSEA = −0.004; ΔSRMR = 0.031), and the scalar model also showed changes within acceptable limits (ΔCFI = −0.004; ΔRMSEA = −0.009; ΔSRMR = 0 .000). Following the criteria proposed by Chen (2007)—ΔCFI ≤ 0 .01; ΔRMSEA ≤ 0.015; and ΔSRMR ≤ 0.030 for metric invariance and ≤ 0.010 for scalar invariance—the results support configural, metric, and scalar invariance across sexes.

To check for the possible influence of age, its predictive value was examined in relation to the total score and in both samples using regression analysis. The results showed that age was not a significant predictor of distress (β = 0.062, *p* = 0.205), suggesting that scores on the scale do not vary systematically according to age in this sample. However, a high percentage of the sample is between 18 and 25 years old, so future studies with larger samples that include a greater proportion of other age groups would be necessary to confirm the findings.

### 3.5 Criterion validity of the EDS

The criterion validity between the three subscales of Loneliness, Meaninglessness and Low Self-worth from the EDS-S, and the scores presented in the *UCLA Loneliness Scale*, the *Purpose In Life Test* and *Rosenberg Self-Esteem Scale* were tested using Spearman correlations. The results shown in Table 7 indicate that the scores of all subscales of the EDS-S are significantly related to the criterion scale scores, negatively all cases because instruments way of scoring.

**TABLE 7:** Correlations between the EDS-S subscales, *UCLA Loneliness Scale, Purpose In Life Test* and *Rosenberg Self-Esteem Scale.*

Subscale	UCLA loneliness scale	Purpose in life test	Rosenberg self-esteem scale
EDS-S loneliness subscale	−0.40**	−0.09	−0.29**
EDS-S meaninglessness subscale	−0.35**	−0.28**	−0.53**
EDS-S low self-worth subscale	−0.30**	−0.32**	−0.35**

**Correlation is significant at level 0.01 (bilateral).

At the subscale level, the Loneliness subscale of the EDS-S showed a negative correlation with the *UCLA Loneliness Scale* (*r* = -0.40, *p* < 0.001), and with the *Rosenberg Self-Esteem Scale (r* = −0.29, *p* < 0.001). The Meaningless subscale was negatively correlated with all criterion measures, showing moderate associations with the *UCLA Loneliness Scale* (*r* = −0.35, *p* < 0.001) and the PIL-10 (*r* = −0.28, *p* < 0.001), and a strong negative correlation with the *Rosenberg Self Esteem Scale* (*r* = −0.53, *p* < 0.001).

Finally, Low Self-worth subscale exhibited significant negative correlations with the *UCLA Loneliness scale (r* = −0.30, *p* < 0.001*)*, the PIL-10 *(r* = −0.32, *p* < 0.001), and the *Rosenberg Self-Esteem Scal*e *(r* = −0.35, *p* < 0.001).

Overall, these findings provide further support for the criterion-related validity of the EDS-S scales.

## 4 Discussion

The aim of the present study was adapt the *Existential Distress Scale* (EDS), renamed the *Existential Distress Scale Spanish version* (EDS-S) *Escala de Angustia Existencial*, Table 8 into Spanish and carry out a preliminary psychometric evaluation in a Spanish non-clinical sample to assess its content and criterion validity and to conduct a factor analysis with an expanded sample compared to the original study by Lo et al. (2016). The results show that the EDS-S is a promising tool for assessing the construct Existential Distress and its three principal components: Loneliness, Low Self-worth and Meaninglessness among Spanish adults. Existential distress is part of the human experience, although it can be identified in clinical contexts, linking it to universal concerns related to meaning, finitude, and isolation (Vos et al., 2015). Within this framework, its assessment in the general population would allow for the establishment of a psychometric baseline from which to analyse its exacerbation in contexts of greater vulnerability.

**TABLE 8:** *Existential Distress Scale Spanish version* (EDS-S); *Escala de Angustia Existencial.* Nos gustaría poder entender lo mejor posible cómo se siente. Para ello le agradeceríamos que nos respondiese a algunas preguntas sobre pensamientos angustiosos. Las preguntas se refieren a si se ha estado sintiendo mal consigo mismo, si se ha sentido solo o si se ha estado cuestionando el sentido de su vida. Por favor, díganos si alguna vez se ha sentido angustiado por alguno de los siguientes pensamientos y si ha sido así, cuánta angustia le ha causado. >Ha sentido angustia al pensar que…?

Item	Ninguna angustia	Angustia Ligera	Angustia Moderada	Gran angustia	Angustia Insoportable
1 ¿Está completamente solo?					
2 ¿Nadie le echará de menos cuando fallezca?					
3 ¿Nadie se preocupa por usted?					
4 ¿Usted es o será una carga para los demás?					
5 ¿No tiene usted nada que ofrecer a los demás?					
6 ¿Usted no importa?					
7 ¿Usted no vale nada?					
8 ¿Su vida está vacía?					
9 ¿Su vida no tiene sentido?					
10 ¿Usted no tiene nada por lo que vivir?					

The EDS-S is an instrument to measure Existential Distress meeting the criteria proposed by Muñiz et al. (2013). The content validity of the instrument as assessed, with a review by experts of the clarity, relevance and coherence of the items. The evidence of content validity is based on the criteria of the American Educational Research Association as one of the five forms of validity stipulated in the Standards for educational and psychological testing (Sireci and Faulkner-Bond, 2014). One of the strengths of this study was the participation of experts in the field of clinical psychology and trauma, where existential distress is part of clinical practice, in addition to experts in research methodology. The retro-translation with a blind translator unfamiliar with the original version ensured the quality of the translation. The separate evaluation of the three dimensions improved the precision of each item and thus favoring accurate assessment (Halek et al., 2017).

Loneliness, meaning in life and self-esteem are considered as principal factors in psychotherapeutic interventions (García-Alandete and Hernández-Jiménez, 2018). The choice of these three dimensions, concentrated in ten items, permits an accurate assessment of existential distress, evaluated in other studies using a specific questionnaire for each dimension. For example, the study by Philipp et al. (2021) evaluated the existential distress of oncology patients and their carers by means of nine questionnaires, including instruments specifically addressing the three factors of the EDS-S. Specifically, these were the Depressive Experiences Questionnaire, with the subscales Dependence and Relationships; the Demoralization Scale; the Structured Interview on Psychological Adjustment and Demoralization to evaluate the same dimensions addressed by the EDS-S. As the results of this research show, the Spanish version of the EDS is a promising tool for evaluating the construct.

The results of the EFA suggest a three-factor construct. While the original study found a single factor, the authors attributed this to a limited sample size. The present study, with a larger sample, provides evidence in favor of a three-factor model. This factorial structure was confirmed through confirmatory factorial analysis (CFA) in the second sample as an independent sample, thereby strengthening the structural validity for the scale.

A relevant issue regarding the factorial structure found is the discrepancy with the results of the study by Lo et al. (2016). While the present study identified a three-factor structure in the non-clinical population—loneliness, low self-worth, and experience of meaninglessness- it is possible that existential distress is expressed differently in serious clinical contexts or terminal illnesses. In healthy people, where the existential threat is more abstract, these dimensions can be differentiated more clearly. However, in situations of extreme crisis or imminent mortality, the existential threat may be experienced in a more global way (Pyszczynski et al., 1999), giving rise to a less differentiated experience. In advanced diseases, existential suffering is often described as a unified experience that integrates meaning, identity, autonomy, and loss (Vehling and Kissane, 2018). In this context, the structure of the construct could be approximated to a more one-dimensional model. From this perspective, the three-dimensional solution found in this study and the original one-dimensional model would not necessarily be contradictory, but rather expressions of the same construct under different levels of severity and population conditions.

The validity of the criterion has been confirmed for the three EDS-S subscales: Loneliness, Low Self-worth, and the Experience of Meaninglessness. Firstly, a negative correlation was identified between the Loneliness dimension of the EDS-S and the *UCLA Loneliness Scale* (Vázquez and Jiménez, 1994), in which higher scores indicate lower degrees of loneliness, in line with the results of similar studies (Lee et al., 2019). Secondly, a negative relationship was identified between the EDS-S dimension ‘Experience of Meaninglessness and “Meaning of Life” on the PIL-10 (García-Alandete, 2014). This again coincides with the results of other studies (Rubio-Belmonte and García-Alandete, 2023). Finally, a significant negative relationship was found between the Low Self-worth dimension of the EDS-S and the results on the *Rosenberg Self-Esteem Scale* (Martín-Albo et al., 2007), in line with the findings reported by Robins et al. (2001). A negative relationship was also observed between *the UCLA Loneliness Scale* and the “Low self-esteem” dimension, consistent with the results of the study by Al Khatib (2012). In addition, a negative relationship was observed between the PIL-10 questionnaire and the “Low self-esteem” dimension (Smedema and Barahona, 2018). It should also be noted that the reported effect sizes indicate partial convergent validity for the three subscales in relation to the criterion tests. The EDS-S can be considered not redundant with existing measures of loneliness, meaning of life, or self-esteem (Shaffer et al., 2015), but rather capturing a construct that is closely related but not equivalent, namely existential distress.

The aim of the study was to evaluate the construct in its pure or baseline state, facilitating the identification of the underlying factor structure of the EDS-Spanish without variables that could bias the strength of the correlations. While this approach ensures high internal validity for this initial validation, the factor structure and intensity of the correlations observed should be interpreted as a reference framework within a healthy population. Is should be noted that validation in healthy samples does not establish suitability for clinical populations. Although the EDS-S appears promising as a brief assessment tool, substantial clinical validation is essential before its implementation in vulnerable groups. Such validation should determine whether the factorial structure is replicated under conditions of extreme distress, whether the items function adequately across the full spectrum of severity, and whether the instrument provides clinically meaningful information in contexts where measurement error may have significant consequences. This study is a necessary preliminary step that allows the instrument to be refined before subjecting it to the stress of actual clinical practice, where the emotional burden on patients requires previously tested tools. In fact, a person’s terminal condition is a variable that could reconfigure the relevance of certain items (Vehling and Kissane, 2018). Therefore, the tool will be useful in clinical populations once convergent validation studies are conducted with terminal patients.

Among the limitations of the study, the use of non-probabilistic snowball sampling may restrict the generalization of the results and have influenced the educational profile of the sample. Likewise, the exclusion of individuals undergoing psychopharmacological treatment, adopted to avoid possible confounding variables in the initial factor structure, limits the scope of the findings. Although the objective was to evaluate the psychometric properties in a normative sample under controlled conditions, these methodological decisions may have affected the variance and internal consistency indices. Future research should include clinical samples and greater socio-educational diversity to examine the stability of the instrument in more heterogeneous contexts.

Furthermore, although the panel of experts was highly qualified in the field of trauma and grief, the absence of professionals currently working in palliative care units is a limitation. This could affect the validity of the content specifically for terminal clinical populations, as some nuances of existential suffering in the immediate proximity of death may not be fully captured from a general or trauma-focused perspective.

Another limitation of the study is the sex imbalance in the first sample, in which 74% of participants were women. The imbalance may affect the external some of the results, given the representativeness of the sample in terms of the general population. However, this imbalance is eliminated with the second sampling, validating the factorial structure through CFA.

This study points to the following prospects: in the short term, priorities should include validation in clinical samples with adequate power (e.g., cancer or palliative care patients), assessment of test-retest reliability over a 2–4-week interval, and direct comparison of factor structure between clinical and non-clinical populations. In the medium term, it is proposed to incorporate known-group validity analyses, sensitivity and specificity analyses to establish empirically derived clinical cut-off scores, as well as tests of measurement invariance across different clinical states presented by patients in future samples. These steps will be considered prerequisites for clinical implementation.

Although the Existential Distress Scale (EDS) was originally designed for clinically oncology settings, this study offers initial validation in a non-clinical Spanish population. While the proposed factorial structure was supported, providing preliminary evidence of structural validity and contributing to the measurement of this construct, this configuration may vary in clinical samples, where existential distress may manifest with greater intensity and complexity. Therefore, this study should be understood as a necessary preliminary step, allowing the instrument to be redefined before its application in real clinical contexts, there emotional burden on patients requires rigorously tested assessment tool.

This study offers the first cross-cultural adaptation and validation of the Existential Distress Scale (EDS) into Spanish, proposing the instrument as an early detection tool. In this context, the EDS-S could be used as a preventive screening tool, facilitating the identification of a person’s level of existential distress and, where appropriate, enabling the development of psychological interventions aimed at reinforcing the meaning of life before the distress evolves into more serious psychopathological conditions or periods of greater vulnerability. Future research should include clinical samples to thoroughly examine the EDS-S’s ability to capture levels of existential distress in this population and to test for factor invariance between clinical and non-clinical groups.

In summary, this research offers a first approach to a valid a reliable version of the EDS in Spanish applicable to non-clinical samples, while establishing an essential frame of reference for future work in clinical contexts, in order to provide support when high levels of existential distress are detected.

## Data Availability

The raw data supporting the conclusions of this article will be made available by the authors, without undue reservation.
